# Helminth Mediated Attenuation of Systemic Inflammation and Microbial Translocation in Helminth-Diabetes Comorbidity

**DOI:** 10.3389/fcimb.2020.00431

**Published:** 2020-08-31

**Authors:** Anuradha Rajamanickam, Saravanan Munisankar, Pradeep A. Menon, Chandrakumar Dolla, Thomas B. Nutman, Subash Babu

**Affiliations:** ^1^National Institute of Health-NIRT-International Center for Excellence in Research, Chennai, India; ^2^Department of Epidemiology, National Institute for Research in Tuberculosis, Chennai, India; ^3^Laboratory of Parasitic Diseases, National Institute of Allergy and Infectious Diseases, National Institutes of Health, Bethesda, MD, United States; ^4^Frederick National Laboratory for Cancer Research, National Cancer Institute, Frederick, MD, United States

**Keywords:** helminths, type 2 diabetes mellitus, systemic inflammation, acute phase proteins, microbial translocation

## Abstract

Type 2 diabetes mellitus (T2DM) is characterized by heightened systemic inflammation and microbial translocation. Whether concomitant helminth infections can modulate this systemic response is unclear. We examined the presence of markers of systemic inflammation (levels of acute phase proteins) and of microbial translocation [levels of lipopolysaccharide (LPS) and its associated products] in T2DM individuals with (*Ss*^+^) or without (*Ss*^−^) *Strongyloides stercoralis* (*Ss*) infection. We also analyzed these parameters at 6 months following anthelmintic treatment in *Ss*^+^ individuals. *Ss*^+^ individuals exhibited significantly diminished levels of alpha-2 macroglobulin, C-reactive protein, haptoglobin and serum amyloid protein A1 compared to *Ss*^−^ individuals and these levels increased significantly following therapy. Similarly, *Ss*^+^ individuals exhibited significantly diminished levels of LPS, sCD14, intestinal fatty acid binding protein, LPS binding protein and endotoxin IgG antibody and most of these levels increased significantly following therapy. Thus, helminth infection is associated with attenuation of systemic inflammation and microbial translocation in T2DM and its reversal following anthelmintic therapy.

## Introduction

Systemic inflammation and microbial translocation are typical characteristics of Type 2 diabetes mellitus (T2DM) and are known to drive T2DM-associated pathology (Donath and Shoelson, 2011; Donath et al., 2019; Tilg et al., 2020). Systemic inflammation in T2DM is often chronic and low grade and is labeled as metabolic inflammation or metaflammation (Hotamisligil, 2017). Several studies have associated T2DM with increased circulating levels of acute phase proteins such as C-reactive protein (CRP), serum amyloid protein A1 (SAA1), haptoglobin as well as cytokines and chemokines (Pickup et al., 1997; Ridker et al., 2000; Pradhan et al., 2001; Festa et al., 2002; Freeman et al., 2002; Thorand et al., 2003). Metaflammation is considered sterile, is driven by non-infectious factors such as nutrients and dietary lipid species, and characterized by endoplasmic reticulum stress and oxidative stress (Fu et al., 2012; Ertunc and Hotamisligil, 2016). Microbial translocation (with resultant metabolic endotoxemia) typically results from intestinal dysbiosis and is associated with increased intestinal permeability and translocation of microbial products into the circulation (Brenchley and Douek, 2012; Levy et al., 2017). It was demonstrated that mice fed a high fat diet exhibited a modified gut microbiome and an influx of bacterial derived lipopolysaccharides (LPS) into the systemic circulation, which, in turn, contributed to an increased susceptibility to T2DM (Cani et al., 2007). Subsequent studies have confirmed the elevation of microbial translocation markers in T2DM compared to healthy controls (Pendyala et al., 2012; Teixeira et al., 2012; Genser et al., 2018). Thus, systemic inflammation and microbial translocation with metabolic endotoxemia are key drivers of T2DM.

Interventions designed to control metabolic inflammation and endotoxemia are attracting a great deal of interest because of their ability to reduce the risk of metabolic diseases and their ability to reduce the pathogenic impact on already developed metabolic diseases (Donath et al., 2019; Tilg et al., 2020). We and others have previously shown that helminth infections are key factors in modulating T2DM-associated processes including insulin resistance, blood glucose and HbA1c levels, and dyslipidemias (Aravindhan et al., 2010; Chen et al., 2013; Hays et al., 2015; Wiria et al., 2015; Rajamanickam et al., 2018). Helminth infections can also modulate systemic inflammation seen in T2DM by down regulating pro-inflammatory cytokines and chemokines (Rajamanickam et al., 2018, 2020).

We postulate, therefore, that helminth infections could also influence systemic inflammation and metabolic endotoxemia by altering the levels of acute phase proteins and microbial translocation markers in T2DM. To this end, we measured the levels of these parameters in those with T2DM, with or without coincident infections with *Strongyloides stercoralis* (*Ss*), a common helminth parasite known to infect 50–100 million people worldwide. We also determined the effect of definitive anthelmintic treatment on the aforementioned parameters in *Ss* infected subjects.

## Materials and Methods

### Ethics Statement

All participants were examined as part of a natural history study protocol (12-I-073) approved by Institutional Review Boards of the National Institute of Allergy and Infectious Diseases (USA) and the National Institute for Research in Tuberculosis (India), and informed written consent was obtained from all participants.

### Study Population

We recruited 118 individuals consisting of 60 clinically asymptomatic *Ss* infected individuals with T2DM (hereafter *Ss*^+^), and 58 individuals with T2DM and no *Ss* infection (hereafter *Ss*^−^) in Kanchipuram District, Tamil Nadu, South India (Table 1) and sample recruitment plan has been shown in Supplementary Figure 1. None had previous anthelmintic treatment, a history of helminth infections or of HIV. These individuals were all recruited from a rural population by screening of individuals for helminth infection by stool microscopy and serology. Follow up was performed at 6 months following treatment. The study groups were matched with respect to age and body mass index. We excluded those individuals who consumed antibiotic or probiotic within the prior 4 weeks, those who had gastrointestinal disease like irritable bowel syndrome, inflammatory bowel disease, history of gastrointestinal cancer or surgical resection, or acute, severe gastrointestinal symptoms and indication of hepatitis B or C virus infection. This was the same study population that was previously used for assessment of metabolic and immune parameters (Rajamanickam et al., 2018, 2020).

**Table 1:** Demographic and biochemical parameters.

	***Ss^**+**^***	***Ss*^**−**^**	***p*-values**
	***n* = 60**	***n* = 58**	
M/F	30/30	30/28	NS
Age	46 (24–63)	45 (22–63)	NS
BMI	28.5 (22.8–32.2)	29.2 (23.3–33.4)	NS
RBG (mg/dl)	179 (140–438)	180.5 (140–198)	NS
HbA1c (%)	8.57 (6.5–12.5)	8.9 (6.5–11.8)	NS
Urea (mg/dl)	19.5 (12.34)	21.9 (11–42)	NS
Creatinine (mg/dl)	0.78 (0.3–1)	0.85 (0.6–1.0)	NS
ALT (U/L)	17.7 (7–60)	22.4 (7–92)	NS
AST (U/L)	27.8 (16–110)	24.7 (11–68)	NS

*The values represent geometric mean and range except for gender and age (which is median and range)*.

### Parasitological Examination and Anthelmintic Treatment

*Ss* infection was diagnosed by the presence of IgG antibodies to the recombinant NIE antigen as described previously (Bisoffi et al., 2014; Buonfrate et al., 2015). Stool microscopy was used to exclude the presence of other intestinal helminth infections. Filarial infection was excluded in all study participants by virtue of being negative in tests for circulating filarial antigen. All INF individuals were treated with a single dose of ivermectin (12 mg) and albendazole (400 mg) and follow-up blood draws were obtained 6 months later.

### Determination of T2DM Status

Diabetes was defined as an HbA1c reading of 6.5% or greater and a random blood glucose of >200 mg/dl, according to the American Diabetes Association criteria.

### Acute Phase Proteins

Plasma levels of alpha-2 macroglobulin (α-2M), C-reactive protein (CRP), haptoglobin, and Serum Amyloid A-1(SAA-1) were measured using a multiplex kit from R&D Systems, Minneapolis, MN, USA. according to the manufacturer's instructions.

### Microbial Translocation Markers

To inactivate plasma proteins, plasma samples were heated to 75°C for 5 min. LPS levels were measured using a limulus amebocyte lysate assay (Cell Sciences Hycult Biotech, Canton, MA, USA) according to the manufacturer's protocol. Commercially available enzyme-linked immunosorbent assay (ELISA) kits were used to measure plasma levels of lipid-binding protein (LBP), endotoxin core antibodies IgG (EndoCAb), intestinal fatty acid binding protein (iFABP) (all Cell Sciences Hycult Biotech), and sCD14 (R&D Systems, Minneapolis, MN, USA).

### Statistical Analysis

Data analyses were performed using GraphPad PRISM 8 (GraphPad Software, Inc., San Diego, CA, USA). Geometric means (GM) were used for measurements of central tendency. Statistically significant differences were analyzed using the non-parametric Mann–Whitney U-test and Wilcoxon matched pair test. Multiple comparisons were corrected using the Holm's correction.

## Results

### Study Population Characteristics

The baseline demographic characteristics and biochemical parameters are shown in Table 1. As shown and as described previously (Rajamanickam et al., 2018, 2020), there were no significant differences in age, sex, BMI or other biochemical parameters between the two groups.

### Diminished Systemic Levels of Acute Phase Proteins in Helminth-Diabetes Comorbidity and Partial Reversal After Anthelmintic Treatment

**Figure 1:**
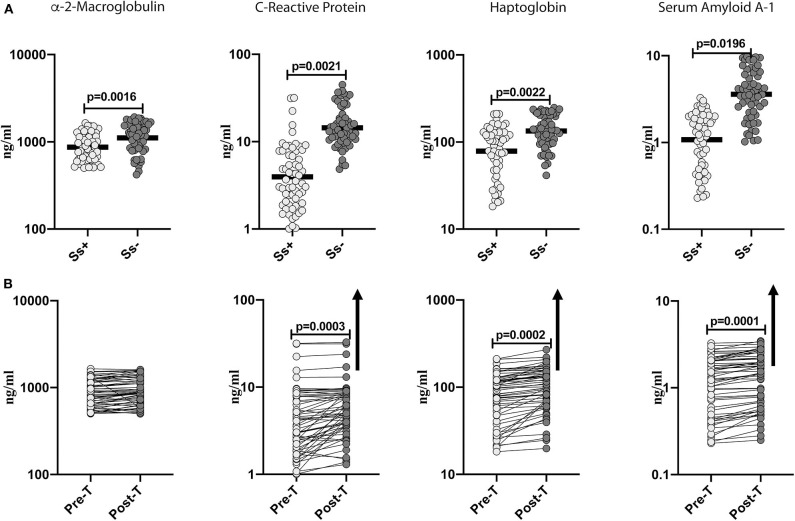
Diminished systemic levels of acute phase proteins in helminth-diabetes comorbidity and partial reversal after anthelmintic treatment. **(A)** Plasma levels of a-2 Macroglobulin, C-reactive protein (CRP), haptoglobin, and Serum Amyloid A-1 (SAA-1) from *Ss* infected [*Ss*^+^] (*n* = 60) or uninfected [*Ss*^−^] (*n* = 58) individuals were measured by multiplex assay. Data are shown as scatter plots with the bar representing the geometric mean. *p*-values were calculated using the Mann-Whitney U-test with Holms correction for multiple comparisons. **(B)** Plasma levels of a-2 Macroglobulin, C-reactive protein (CRP), haptoglobin, and serum amyloid proteins (SAA), from *Ss*-infected individuals at pre-treatment [pre-T] (*n* = 60) and at 6 months following treatment [post-treatment (post-T)] were measured by multiplex assay. The arrow shows the directionality of the significance. *p*-values were calculated using the Wilcoxon matched pair test.

To determine the effect of *Ss* infection on systemic inflammation in T2DM, we measured the levels of acute phase proteins (α-2M, CRP, haptoglobin and SAA-1) in *Ss*^+^ and *Ss*^−^ individuals. As shown in Figure 1A, the levels of α-2M (GM of 868.6 ng/ml in *Ss*^+^ compared to 1,109 ng/ml in *Ss*^−^; *p* = 0.0016), CRP (GM of 3.962 ng/ml in *Ss*^+^ compared to 5.368 ng/ml in *Ss*^−^; *p* = 0.0021), haptoglobin (GM of 78.7 ng/ml in *Ss*^+^ compared to 134.1 ng/ml in *Ss*^−^; *p* = 0.0022) and SAA-1 (GM of 1.018 ng/ml in *Ss*^+^ compared to 3.593 ng/ml in *Ss*^−^; *p* = 0.0196) were significantly lower in *Ss*^+^ compared to *Ss*^−^ individuals. Next, we wanted to determine the effect of anthelmintic treatment on the acute phase proteins in *Ss*^+^ individuals at 6 months following anthelmintic treatment. As shown in Figure 1B, the post-treatment levels of CRP (percentage increase of 30%; *p* = 0.0003), haptoglobin (percentage increase of 21%; *p* = 0.0002) and SAA-1 (percentage increase of 15%; *p* = 0.0001) were significantly increased when compared to pre-treatment levels. Thus, *Ss* infection is associated with diminished systemic inflammation in individuals with T2DM and a partial reversal following treatment.

### Diminished Systemic Levels of Microbial Translocation Markers in Helminth-Diabetes Comorbidity and Partial Reversal After Anthelmintic Treatment

**Figure 2:**
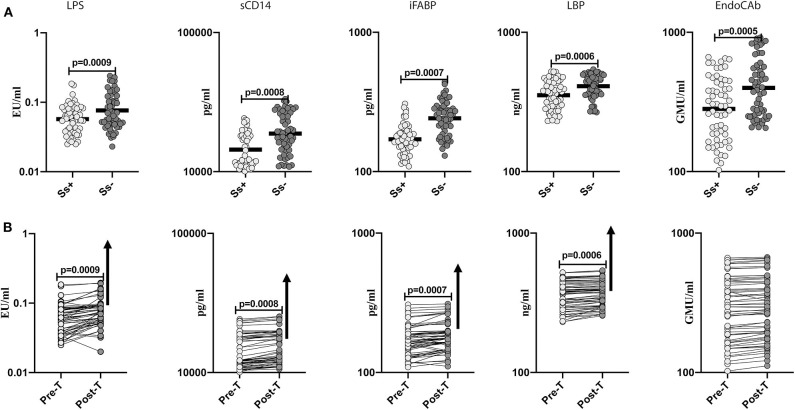
Diminished systemic levels of microbial translocation markers in helminth-diabetes comorbidity and partial reversal after anthelmintic treatment. **(A)** Plasma levels of lipopolysaccharide (LPS), soluble CD14 (sCD14), intestinal fatty acid binding protein (iFABP), lipid binding protein (LBP), and endotoxin core antibody IgG (EndoCAb), from *Ss* infected [*Ss*^+^] (*n* = 60) or uninfected [*Ss*^−^] (*n* = 58) individuals were measured by ELISA. Data are shown as scatter plots with the bar representing the geometric mean. *p*-values were calculated using the Mann-Whitney U-test with Holms correction for multiple comparisons. **(B)** Plasma levels of lipopolysaccharide (LPS), soluble CD14 (sCD14), intestinal fatty acid binding protein (iFABP), lipid binding protein (LBP), and endotoxin core antibody IgG (EndoCAb) from *Ss*-infected individuals at pre-treatment [pre-T] (*n* = 60) and at 6 months following treatment [post-treatment (post-T)] were measured by ELISA. The arrow shows the directionality of the significance. *p*-values were calculated using the Wilcoxon matched pair test.

To determine the effect of *Ss* infection on intestinal dysbiosis and metabolic endotoxemia in T2DM, we measured the levels of microbial translocation markers (LPS, sCD14, iFABP, LBP and EndoCAb) in *Ss*^+^ and *Ss*^−^ individuals. As shown in Figure 2A, the levels of LPS (GM of 0.057 EU/ml in *Ss*^+^ compared to 0.076 EU/ml in *Ss*^−^; *p* = 0.0009), sCD14 (GM of 1,441 pg/ml in *Ss*^+^ compared to 1,879 pg/ml in *Ss*^−^; *p* = 0.0008), iFABP (GM of 170.7 pg/ml in *Ss*^+^ compared to 241.4 pg/ml in *Ss*^−^; *p* = 0.0007), LBP (GM of 353.8 ng/ml in *Ss*^+^ compared to 411.2 ng/ml in *Ss*^−^; *p* = 0.0006) and EndoCAb (GM of 282.1 GMU/ml in *Ss*^+^ compared to 399.6 GMU/ml in *Ss*^−^; *p* < 0.0001) were significantly lower in *Ss*^+^ compared to *Ss*^−^ individuals. Next, we wanted to determine the effect of anthelmintic treatment on the microbial translocation markers in *Ss*^+^ individuals at 6 months following anthelmintic treatment. As shown in Figure 2B, the post-treatment levels of LPS (percentage increase of 24%; *p* = 0.0009), sCD14 (percentage increase of 6%; *p* = 0.0008), iFABP (percentage increase of 7%; *p* = 0.0007), and LBP (percentage increase of 5%; *p* = 0.0006) were significantly increased in *Ss*^+^ individuals when compared to pre-treatment levels. Thus, *Ss* infection is associated with diminished microbial translocation and metabolic endotoxemia in individuals with T2DM and a partial reversal following treatment.

## Discussion

Our study examined the regulation of acute phase proteins and microbial translocation markers in helminth—diabetes co-morbidity and the effect of anthelmintic treatment on these markers. Helminth interactions with metabolic disorders has garnered attention recently due to the finding that coexistent helminth infections could mediate beneficial effects on the metabolic dysfunction in these diseases (De Ruiter et al., 2017; Van Der Zande et al., 2019). Thus, while there exists an inverse relationship between helminth infections and metabolic disorders such as T2DM, there are also reports that helminths might potentially confer protection against insulin resistance, dyslipidemias and overt diabetes pathology (Aravindhan et al., 2010; Chen et al., 2013; Hays et al., 2015; Wiria et al., 2015; Rajamanickam et al., 2018). In fact, a clinical trial has been proposed to examine the safety and tolerability of hookworm infection in individuals with metabolic disease (Pierce et al., 2019). Thus, it becomes important to unravel the mechanisms behind the helminth—metabolic disorder interaction. We have used the presence of *Ss* infection in T2DM individuals to study one aspect of this interaction (i.e., the effect on systemic inflammation and metabolic endotoxemia), both of which are hallmarks of T2DM.

Elevations in acute phase proteins is a prototypical finding in the chronic, systemic inflammation that characterizes metabolic disorders, including T2DM (Pickup et al., 1997; Ridker et al., 2000; Pradhan et al., 2001; Festa et al., 2002; Freeman et al., 2002; Thorand et al., 2003). Unresolved endoplasmic reticulum stress and oxidative stress are key drivers of an inflammatory responses in metabolic dysregulation (Fu et al., 2012; Ertunc and Hotamisligil, 2016). Various markers of inflammation especially CRP have been shown to be associated with a higher predisposition to T2DM and consequent cardiovascular disease (Esser et al., 2014). Thus, a meta-analysis of 22 studies showed that elevated CRP levels were associated with increased risk of T2DM (Wang et al., 2013), and another meta-analysis of 52 prospective studies showed that elevated CRP is a predictor of cardiovascular events (Emerging Risk Factors et al., 2012). α-2M, SAA1 and haptoglobin are markers associated with diabetic complications and duration of disease, and therefore reduction in these acute phase proteins might potentially be a mechanism of lowering the occurrence of complications in T2DM (Jonsson and Wales, 1976; McMillan, 1976; Mackellar and Vigerust, 2016). Our data clearly reveals an association of lower levels of acute phase proteins in the presence of *Ss* infection and a partial reversal following anthelmintic treatment. Thus, our study elucidates a novel mechanism by which concomitant helminth infection might potentially modulate pathology in T2DM.

Several studies have reported the presence of intestinal dysbiosis and increased intestinal barrier permeability in T2DM (Mooradian et al., 1986; Pendyala et al., 2012; Qin et al., 2012; Cotillard et al., 2013; Thaiss et al., 2016). Other studies have clearly revealed a role for endotoxemia as a driver of metabolic diseases (Gomes et al., 2017; Tilg et al., 2020). Endotoxemia and its associated biomarkers (elevated LPS and LBP) were linked to increased risk of obesity and T2DM and correlated with disease exacerbation in humans (Mehta et al., 2010; Sun et al., 2010; Pussinen et al., 2011; Camargo et al., 2019). Furthermore, LPS administration to mice and healthy humans results in systemic and adipose tissue inflammation and insulin resistance, confirming that LPS affects insulin sensitivity (Mehta et al., 2010). Other products of microbial translocation including iFABP, a marker of gut permeability (Cox et al., 2017), sCD14 (Shitole et al., 2019), LBP (Sun et al., 2010; Cox et al., 2017; Sakura et al., 2017), and EndoCAb (Barengolts et al., 2019) are also associated with pathology in T2DM. Our data reveal a novel association of diminished metabolic endotoxemia and intestinal permeability in the presence of *Ss* infection. Although, *Ss* infection by itself is known to promote microbial translocation (Rajamanickam et al., 2017), its influence in the presence of a diabetic environment appears to be one of downmodulation and this could be due to differential interaction of the helminth infection with the gut microbiota in the context of diabetes mellitus. More conclusive proof that *Ss* drives this downregulated microbial translocation comes from our data on the significant reversal of this downmodulated response following anthelmintic treatment. Thus, chronic helminth infection acts as an immunomodulator in T2DM by dampening metabolic endotoxemia.

While numerous studies have recently examined the epidemiological and clinical association of helminth infection and T2DM, very few studies have directly addressed the underlying mechanism behind this interaction. Modulation of the inflammatory milieu would be expected to be similar in other helminth infections since previous studies have clearly delineated an effect of hookworm and Schistosoma infections on metabolic syndrome/diabetes mellitus (Mishra et al., 2014; Tang et al., 2017; Toniolo et al., 2019). We have previously shown (using the same patient cohort) that concomitant *Ss* infection modulates systemic cytokine, chemokine, adipokine, and hormonal responses that favor protection from insulin resistance and pancreatic beta cell exhaustion. In the current study, we expand upon this to demonstrate a beneficial effect of helminth infection on the systemic inflammatory milieu, intestinal dysbiosis and metabolic endotoxemia, that is highly characteristic of T2DM. Further elucidation of the molecular pathways of this protective effect should provide greater insight into this interesting field of multi-morbidity.

## Data Availability Statement

All datasets presented in this study are included in the article/Supplementary Material.

## Ethics Statement

All participants were examined as part of a natural history study protocol (12-I-073) approved by Institutional Review Boards of the National Institute of Allergy and Infectious Diseases (USA) and the National Institute for Research in Tuberculosis (India), and informed written consent was obtained from all participants.

## Author Contributions

SB: conceptualization, project administration, supervision, and writing and/or original draft. AR and SB: data curation, formal analysis, validation, and visualization. SB and TN: funding acquisition, software, and writing and/or review and editing. AR and SM: investigation and methodology. CD and PM: resources. All authors contributed to the article and approved the submitted version.

## Conflict of Interest

The authors declare that the research was conducted in the absence of any commercial or financial relationships that could be construed as a potential conflict of interest.

## Supplementary Material

Supplementary Figure 1 Sample recruitment flowchart. Participants were screened for T2DM and T2DM positive participants were further tested for *Strongyloides stercoralis (Ss)* infection. Based on study inclusion criteria, participants were recruited for the study *Ss*^+^ (*n* = 60) and *Ss*^−^ (*n* = 58).
